# Current concepts and future approaches to vestibular rehabilitation

**DOI:** 10.1007/s00415-015-7914-1

**Published:** 2016-04-15

**Authors:** Fredrik Tjernström, Oz Zur, Klaus Jahn

**Affiliations:** Department of Oto-Rhino-Laryngology, Head and Neck Surgery, Clinical Sciences, Lund University, Lund, Sweden; Department of Physical Therapy, Ben Gurion University of the Negev, Beer Sheva, Israel; The Israeli Center for Treating Dizziness and Balance Disorders, Raanana, Israel; German Center for Vertigo and Balance Disorders, Ludwig-Maximilians-University of Munich, Munich, Germany; Department of Neurology, Schön Klinik Bad Aibling, Kolbermoorer Str. 72, 83043 Bad Aibling, Germany

**Keywords:** Adaptation, Habituation, Sensory reweighting, Vestibular rehabilitation

## Abstract

Over the last decades methods of vestibular rehabilitation to enhance adaptation to vestibular loss, habituation to changing sensory conditions, and sensory reweighting in the compensation process have been developed. However, the use of these techniques still depends to a large part on the educational background of the therapist. Individualized assessment of deficits and specific therapeutic programs for different disorders are sparse. Currently, vestibular rehabilitation is often used in an unspecific way in dizzy patients irrespective of the clinical findings. When predicting the future of vestibular rehabilitation, it is tempting to foretell advances in technology for assessment and treatment only, but the current intense exchange between clinicians and basic scientists also predicts advances in truly understanding the complex interactions between the peripheral senses and central adaptation mechanisms. More research is needed to develop reliable techniques to measure sensory dependence and to learn how this knowledge can be best used—by playing off the patient’s sensory strength or working on the weakness. To be able using the emerging concepts, the neuro-otological community must strive to educate physicians, physiotherapists and nurses to perform the correct examinations for assessment of individual deficits and to look for factors that might impede rehabilitation.

## Introduction

Vestibular rehabilitation is a broad concept that not only implies compensation training after a vestibular lesion or disease, but also postural training and compensation in other causes of vertigo, dizziness or general unsteadiness. It covers a wide clinical area, in which central nervous adaptation mechanisms to a sensory loss or mismatch are vital. To guide training, it is equally important using the right tools for assessments of sensory function, sensory weighting and identification of factors that might protract compensation. The need for knowledge of methods and concepts for vestibular and postural rehabilitation should not be underestimated. In developed countries, the cost associated with falls is high and with an aging population it is a mounting problem that will demand huge resources from hospitals, as well as from the community. In Sweden (<10 M inhabitants), the annual cost from falls (2009) amounted to 1.4 billion euro, of which almost 500 million were direct costs and 900 million were related to deterioration in quality of life. These costs are expected to increase to approximately 2.2 billion euro by 2050 if the situation continues to develop at its present rate [1]. Abnormal performance on balance tests is the second most important intrinsic predictor for falls in elderly [2], and 35 % of people above 40 years of age have vestibular dysfunction [3].

Acute vestibular loss is the most studied condition and compensation follows certain well-defined steps. The first compensation process consists of central *vestibular adaptation*, in which the symptoms of acute vestibular loss (spontaneous nystagmus, head and ocular tilt, postural disequilibrium, altered vestibulo-ocular and spinal reflexes [4]), are diminished within the first week due to cerebellar modulation (inhibition [5]) of the initial asymmetric activity of the vestibular nuclei [6, 7]. The symptoms gradually resolve and the spinal imbalance normalizes and behavioral recovery is initiated [4, 8] in a process where plastic changes of the activity of the vestibulocerebellum are essential [7, 9]. However, the dynamic loss of vestibular reflexes persists and remains functionally inadequate and asymmetric [10]. It should be mentioned that the rebalancing of the vestibular nuclei is not only a consequence of cerebellar inhibition but also of direct cellular and synaptic adaptations within the vestibular nuclei. This is important as must drugs used in the early phase of an acute vestibular syndrome do interact directly with binding site in the vestibular nuclei (e.g., histaminergic, GABAergic, glutamatergic receptors). The second stage consists of *sensory reweighting*, in which the importance of each sensory system (vision, somatosensation and vestibular) for maintaining postural control is reevaluated and changed [11, 12]. The sensory systems overlap in terms of detection of motion frequency and share some properties of the feed-forward mechanisms involved in maintaining postural control. In that sense, the normal postural control system is redundant and sensory systems are able to replace each other. The last stage is continuous sensory calibration and formation of internal feed-forward models generated by everyday activities and from specific postural training.

**Fig. 1:**
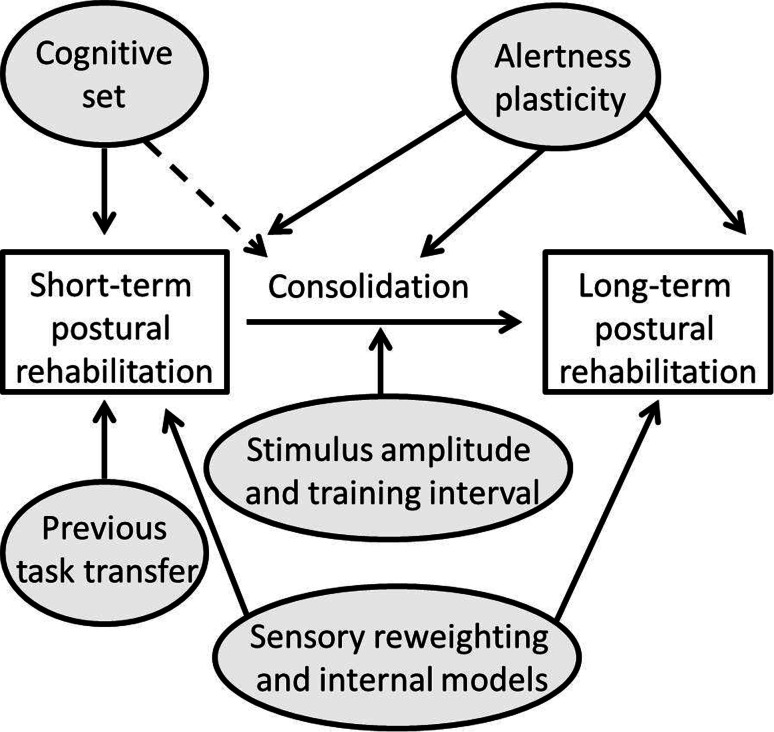
Postural learning or rehabilitation follows the same processes as general memory formation, i.e., from short-term to long-term learning through the process of consolidation, in which the training experiences are reprocessed during inactivity [14]. The process is affected by the cognitive state of mind (e.g. anxiety [17]) and by central nervous plasticity [59, 60] and impeded by reduced alertness from sedatives [61] or sleep deprivation [62]. When designing rehabilitation exercises, it is important to take into account that one exercise might affect succeeding exercises [63], and that the exercises themselves must be sufficiently challenging to promote learning [16, 63]. Learning might be through sensory reweighting or formation of internal models, i.e., motor programs whose output consists of preformed neuromuscular strategies activated automatically or voluntarily in given situations (anticipated movements) [64]

Rehabilitation therapy consists of exercises that train the sensory systems to interact and be integrated within the central nervous system to provide the correct spatial cues for position as well as for head and body motion. Today we know that vestibular training follows the same kind of Hebbian learning as general memory formation [13], where the experiences from exercises are consolidated during inactivity and thus strengthened (Fig. 1) [14, 15]. The central nervous system processes or plasticity can be enhanced by difficult or challenging exercises [16] for many, but not all patients. In patients with anxiety-related dizziness, the central adaptation processes as well as the sensory-integration processes within the brain are fundamentally affected. In these cases, difficult postural exercises might be counter-productive [17].

### Assessing sensory strategies

To plan the right rehabilitation strategy, it is crucially important to be able to correctly assess sensory function and weighting of the dizzy patient and thus the sensory strategy of an individual. All rehabilitation programs aim at training the remaining senses to strengthen their interactions, as well as their integration in the brain balance network. For most patients, reweighting is beneficial, but some become over-reliant on a certain sensory system and thus suffer from sensory mismatch [18]. The most flagrant example of this maladaptation is the concept of “visual vertigo” or secondary “phobic postural vertigo” [19], i.e., that visual stimuli induce illusions of self-motion or an erroneous spatial orientation [20–22]. Accordingly, in a demanding environment, it is significantly more difficult for these patients to ignore visual stimulations even when they are asked to focus on a stationary target [23]. Although the concept of sensory reweighting is widely accepted, it is difficult to measure or even assess reliably. The most widely used clinical test for sensory dependence is the Romberg test, (comparing postural sway when the eyes are opened vs. closed). In posturography, a ratio can be calculated between the tests which has been labeled both as an index of visual dependence [12] and as somatosensory dependence [24]. One problem with calculating ratios is that variations or changes could be artifacts from small variations in either the denominator or the numerator. Because of the sensory overlap, the innate ability to change postural strategy, at least in easy postural tasks, and adaptation to difficult postural tasks [14, 15] (the subjects could have more or less postural sway in any condition), yields low consistency in the sensory profile of an individual when tested repeatedly [25].

The sensory organization test (SOT) in Equitest posturography is often used to appreciate individual sensory weighting in the postural control system [24]. The SOT consists of a series of postural challenges of increasing complexity, which have been shown to correspond to sensory deficits. Ratios from the different conditions are often labeled as indices of vestibular, visual and somatosensory weighting. Measuring different conditions during stance and gait often helps to identify factors contributing to unsteadiness [26, 27]. However, these measures have not been validated between labs and are therefore not available everywhere. This makes the results difficult to interpret when it comes to sensory weighting. The tests also induce central adaptation, which stresses the need for additional methods to assess sensory reweighting [15]. Other evaluations for appreciating visual dependency are the rod and frame and rod and disk tests [28, 29]. The results from these tests have rarely been compared to posturography measurements, but there are definite correlations between the tests in patients suffering from visual vertigo, that validates the rod and disk test, at least in the subgroup of dizzy patients [30].

### Factors impeding rehabilitation

Factors that protract compensation and prolong subjective symptoms have to be recognized early to prevent development of chronic unsteadiness. It is important to screen for risk factors for a prolonged course of compensation after acute vestibular lesions as for example advanced age, medication, microvascular brain lesions and preexisting sensory deficits. Concepts like “Functional dizziness”, persistent postural–perceptual dizziness (PPPD) [31], phobic postural vertigo (PPV), and chronic subjective dizziness (CSD) have helped to advance knowledge of the relationship between anxiety/depression and dizziness. Patients suffering from one of these overlapping syndromes have been shown to interpret sensory cues incorrectly [32]. By doing so the postural control system in unchallenged conditions is already extended to the point that they do not learn or adapt to postural training [17, 33]. These factors compromise rehabilitation. It has also been shown that if the diagnosis is delayed, the condition will be harder to cure [34]. Various treatments have been tried, e.g., regular vestibular exercises combined with cognitive behavioral therapy. Although the short-term results are promising, the long-term result is less convincing so far [35]. Retrospective studies on anxiolytic drugs and antidepressants have shown beneficial effects, especially if the original triggering disease was vestibular [36]. Considering the incidence of the condition and the ensuing functional disability, randomized controlled studies on treatment of phobic dizziness and vertigo are urgently needed. Muscular pain and tension are important conditions that co-exist with dizziness and result in protracted disability, as well as distorted spatial orientation and dizziness [37–39]. Chronic pain also results in central sensitization [40], which is very much in line with motion sensitivity observed in dizzy patients.

### Future aspects of vestibular rehabilitation

In all probability, the health care systems, at least in developed countries, will not receive greater funding for preventing falls or for rehabilitating dizzy patients. With the aging of the population, we will thus need to accomplish more for less money. Engaging in physical activity prior to a sensory loss is beneficial for rehabilitation [41], which implies that the entire population should be encouraged to be physically active and that the beneficial aspects of activities probably outweigh the cost of subsidization. As physicians and physiotherapists, we also need to anticipate when rehabilitation will be needed and pre-habilitate patients who might suffer from sensory loss (e.g., before vestibular surgery or other interventions that result in vestibular deafferentation, but also in chronic vestibular disorders such as Menière’s disease) [42].

For most patients, standard exercises after a vestibular loss will be sufficient to achieve subjective compensation. In the future, new sets of exercises will also focus on creating a mismatch between the sensory systems to which patients have to adapt [43]. However, we need a greater variety of exercises that meet the demands of the individuals’ sensory strategy. Currently, most exercises involve static visual cues that are used for visual recalibration of spatial and postural orientation. However, every day activities involve eye tracking of moving targets, while at the same time moving the head and body, which highlights the need for developing suitable, sufficiently complex exercises [44, 45]. Ideally, the individual patient should be mapped for his/her sensory strategy and then treated with different subsets of exercises that challenge him or her [46, 47]. Additional research for individualization of rehabilitation is of the utmost importance. Despite the wide acceptance of the hypothesis of sensory reweighting, virtually no research is being conducted to determine whether exercises should be designed to augment an individual’s sensory strength (e.g., visually dependency) or to boost the weakness (vestibular loss). With new methods of measuring vestibular function, i.e., the quantitative head impulse test [48], phenomena like the covert saccade have been discovered in patients with a deficient vestibulo-ocular reflex [49]. A covert saccade is an eye saccade that occurs during the head movement in the head impulse test, mimicking the vestibulo-ocular reflex. Its exact origin is unknown, although it has been suggested to involve a cervico-ocular reflex or an anticipatory strategy for the eyes to focus on the target and not wait for the system to react to the nonexistent vestibular information [50]. The presence of covert saccades suggests that the vestibular dysfunction has been compensated and eye-head coordination anticipates movements in real life situations. Thereby, an alternate strategy has been formed, which could be interpreted as better compensation, or compensation beyond vestibular only strategies.

The future will increasingly involve smart-phone applications for exercises, monitoring and possibly for diagnostic purposes, as well as institutional and home-based virtual reality exercise programs [51, 52]. The mobile applications are already developed and will possibly soon be available for treatment outside of the laboratory or the care giver’s office. The gyro that is built in most smart-phones can be used for feed-back guided postural training in virtual reality, much in the same way as larger devices are able to do today (e.g., Wii-Fit^®^). The applications will rapidly be further developed and used for postural feedback training in real life situations. For instance the concept of geo-tagging could be applied for these programs, i.e., in “real” reality walk a specific route, fraught with postural and sensory challenges that would be beneficial for the particular individual. Applications have also the great advantage to monitor progress or lack thereof at a distance and will likely be cost beneficial.

Patients with balance problems and difficulties walking due to vestibular deficits might also benefit from recent developments in robot-assisted gait training [53–55]. Exoskeletons with and without treadmill support (e.g., Hocoma Lokomat^®^, Eksobionics Ekso GT^®^) allow high repetition rates and are therefore beneficial to re-learn automated gait patterns. End-effector based systems (e.g., Ectron Geo^®^) can simulate any gait pattern and allow the training of stair climbing and other demanding conditions.

Lastly, replacement and modulation of the deficient vestibular system are emerging areas of research. Modulation of activity of vestibular afferents, namely by noisy galvanic stimulation is a promising area, not only in vestibular rehabilitation. It has been shown that information processing in a variety of sensory systems can be enhanced by adding an imperceptible amount of noise to the sensor afferents [56]. The rationale behind this is the stochastic resonance in sensory systems. Accordingly it was shown that subthreshold noise input to the vestibular system is able to improve postural balance in healthy subjects and patients with vestibular loss [57]. It can be expected that neuro-modulation techniques like noisy galvanic vestibular stimulation will increasingly be used in the near future to enhance sensory functions. Vestibular implants have been developed using different approaches to restore the lacking vestibular function. Current results demonstrate that electrical stimulation with implants is a safe and can activate the vestibular system [58]. However, it is not clear which patients will have a benefit from surgery and to which extent relevant aspects like quality of life, functioning, and participation will improve. Further, in a heterogeneous patient population with very different etiologies and disease durations the right patients have to chosen for invasive procedures.

**Fig. 2:**
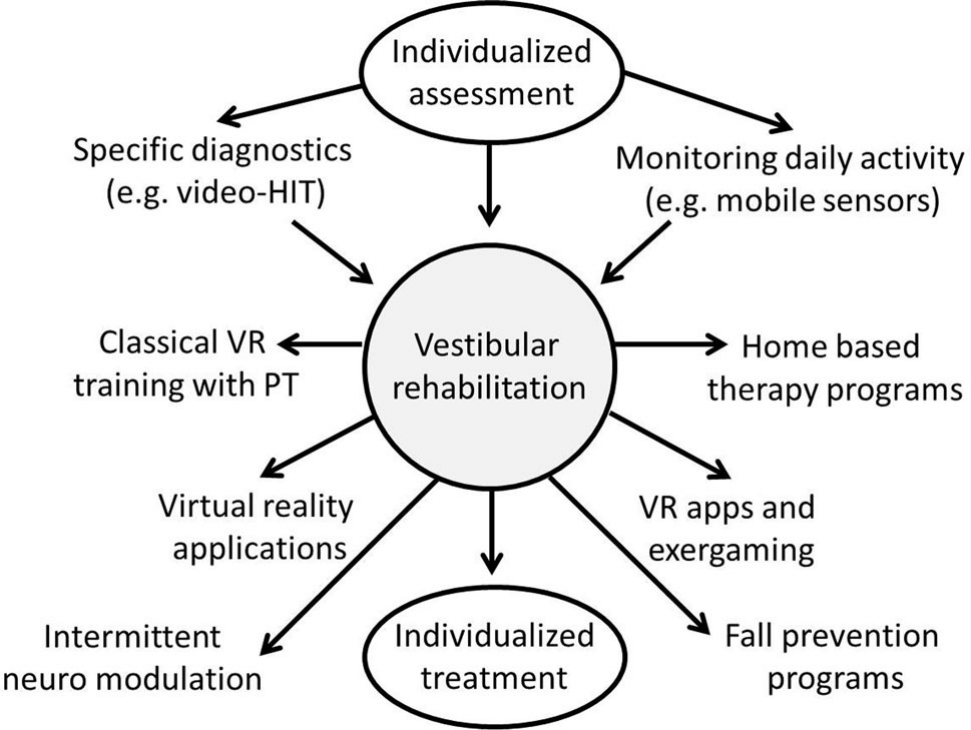
Summary of trends in vestibular rehabilitation. Individualized assessment of deficits is the prerequisite for individualized treatment. New technologies and modern assessment will support the development of disease specific programs. *HIT* head impulse test, *PT* physical therapist, *VR* vestibular rehabilitation

In summary, we expect the programs of vestibular rehabilitation to become more specific in the future. The interaction between clinicians and basic scientists, as well as high standards for the professional education of physical therapists will ensure the development of new rehabilitation programs based on pathophysiological concepts. Emerging technologies including mobile sensors, on demand training using mobile apps, training in virtual environments, the use of neuro-modulation, and the development of vestibular implants will further enlarge the spectrum of therapies available for dizzy patients (Fig. 2).
